# Brillouin scattering-induced rogue waves in self-pulsing fiber lasers

**DOI:** 10.1038/srep45868

**Published:** 2017-04-04

**Authors:** Pierre-Henry Hanzard, Mohamed Talbi, Djouher Mallek, Abdelhamid Kellou, Hervé Leblond, François Sanchez, Thomas Godin, Ammar Hideur

**Affiliations:** 1 CORIA UMR 6614, Normandie Université, CNRS-INSA-Université de Rouen, Saint Etienne du Rouvray, France; 2 Laboratoire d’Electronique Quantique, Université des Sciences et de la Technologie H. Boumediene, Algeria; 3 Laboratoire de Photonique d’Angers EA 4464, Université d’Angers, Angers, France

## Abstract

We report the experimental observation of extreme instabilities in a self-pulsing fiber laser under the influence of stimulated Brillouin scattering (SBS). Specifically, we observe temporally localized structures with high intensities that can be referred to as rogue events through their statistical behaviour with highly-skewed intensity distributions. The emergence of these SBS-induced rogue waves is attributed to the interplay between laser operation and resonant Stokes orders. As this behaviour is not accounted for by existing models, we also present numerical simulations showing that such instabilities can be observed in chaotic laser operation. This study opens up new possibilities towards harnessing extreme events in highly-dissipative systems through adapted laser cavity configurations.

Extreme-amplitude events and rare instabilities are observed for a decade in various optical systems^1^,^2^. Due to the analogy between nonlinear wave propagation in optical waveguides and hydrodynamics, these (temporally or spatially) highly-localized events are termed “optical rogue waves”. Such a terminology was first used in 2007 in the experimental study by Solli *et al*.^3^, reporting the observation of rogue events and highly-skewed intensity distributions in fibre supercontinuum (SC) generation. Since then, various extreme behaviors have been investigated in conservative or weakly-dissipative systems governed by the nonlinear Schrödinger equation (NLSE). In this context, dynamics is dominated by modulation instability (MI) and exact solutions of the NLSE exist in the form of breathers or solitons on finite background (SFB) such as Akhmediev breathers^4^, Kuznetsov-Ma solitons^5^ or Peregrine solitons^6^. These solutions are often referred to as prototypes of rogue waves even if collisions between elementary SFB seem to be a more appropriate way for identifying them^7^. The rich underlying physics of these noise-seeded processes has thus motivated many experimental and numerical studies in the frame of SC generation and soliton propagation^1^ and various techniques have been employed for controlling rogue waves^8^,^9^. As opposed to the above-mentioned single-pass emergence of rogue waves in conservative systems, such extreme events have also been reported in highly-dissipative systems such as laser oscillators, and the term “rogue wave” has also generally been used even if the oceanic analogy is no longer valid. There is however a keen interest from the scientific community about the dynamical behavior of such systems and the underlying physical processes involved. Long-tailed statistics and highly-localized temporal structures have thus been reported experimentally and numerically in various laser configurations such as Raman fiber lasers^10^,^11^,^12^, optically injected semiconductor lasers^13^, laser diodes with optical feedback^14^, chaotic multipulse mode-locked fiber lasers^15^,^16^,^17^, normal dispersion fibers lasers^12^,^18^ and even standard Ti:Sapphire lasers^19^. Rogue events can however be expected in some of these systems due to the complex noise-driven processes intrinsically at the origin of laser operation. In some specific cases, rogue behaviors have been attributed to the direct transfer of pump instability to the laser output dynamics^20^. In addition, due to the interplay between stochastic processes and deterministic dynamics^13^,^21^, dissipative rogue waves can somehow be predicted, controlled and inhibited in some laser systems^22^,^23^.

Here, we focus on the generation of extreme events in passively Q-switched fiber lasers based on nonlinear processes such as stimulated Brillouin (SBS) and Raman (SRS) scatterings. These mechanisms have been extensively studied in last decades^24^,^25^,^26^,^27^,^28^,^29^ and in particular, the exploitation of SBS backscattering as a passive Q-switching mechanism^24^,^29^ or for pulse compression in actively Q-switched lasers and amplifiers has allowed for the generation of short pulses with high peak powers^25^,^30^,^31^. More recently, SBS has been exploited to improve the Q-value of a random laser^32^ thus leading to the first demonstration of a Q-switched random fiber laser^33^. However, the intrinsic stochastic nature of SBS can lead to unstable pulsed operation characterized by the random emission of giant transient pulses which can cause irreversible damages to the fiber laser system itself. This has severely limited the proliferation of this laser technology. Understanding in details the processes involved in the generation of such giant pulses has attracted significant interest in last decades without reaching a solution for controlling their dynamics. Moreover, the nature of these random events and their links with the physical processes is still not clearly established. Using a simple experimental setup along with detailed statistical analysis, in combination with numerical simulations, we show for the first time to our knowledge that for specific cavity parameters the interplay between laser dynamics and SBS can result in the generation of extreme events characterized by highly-skewed intensity distributions.

## Results

### Experimental results

**Figure 1:**
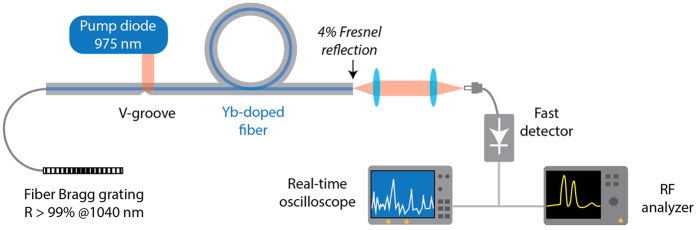
Experimental setup. Laser oscillation is achieved via a highly-reflective fiber Bragg grating centered at 1040 nm on one side and reflection on the fiber facet on the other side.

A rather simple setup, similar to the one presented in ref. 27 and shown in Fig. 1, has been used to investigate for the formation of optical rogue waves in a self-pulsing fiber laser system. The laser consists in a side-pumped ytterbium-doped double-clad (DC) fiber amplifier inserted in a high-loss linear cavity. The active medium (4 m-long Yb-doped fiber) is pumped by a laser diode emitting at *λ* = 975 nm using the V-groove technique to couple up to 70% of the pump power into the inner cladding (125 × 125 *μ*m^2^ square). The core diameter is 7 *μ*m (NA∼0.12). Two segments of single mode fibers (SMF) are spliced at both ends of the doped fiber and act as spatial filters for the 975 nm pump signal. A highly-reflective fiber Bragg grating (FBG, *R* > 99%, 200 pm spectral width around 1040 nm, IxFiber, Inc) fusion-spliced at one end and the right-angle-cleaved fiber at the other end ensure laser oscillation at *λ*_*l*_ = 1040 nm.

**Figure 2:**
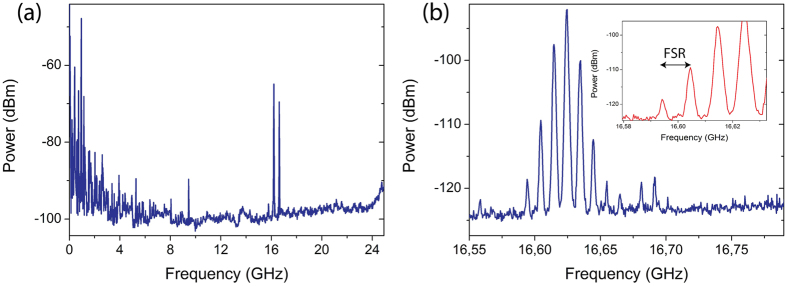
RF spectra showing the presence of SBS around 16.2 and 16.6 GHz. (**a**) Long span RF spectrum. (**b**) Zoom on the 16.6 GHz signal modulated at the FSR (10 MHz) as shown in the inset.

The output signal is analyzed using a high-speed photodetector (25 GHz bandwidth) and a 23 GHz real-time oscilloscope with 512 MPts/channel capacity allowing to record time series up to 10 s with a 500 ps/point resolution in order to have sufficient statistics. Contrary to previous studies involving ultrafast lasers, the timescales considered in our system do not require any real-time characterization techniques^3^,^34^ as rogue pulses can be detected using standard fast electronics. Such measurements however come with the drawback of heavy data files and long data processing times. Here, SBS in self-pulsing laser operation can be evidenced by the presence of a Stokes wave shifted by 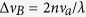 from the pump, where n, *v*_*a*_ and *λ* are respectively the refractive index, the acoustic velocity and the pump wavelength. In the SMF fibers Δ*v*_*B*_ is calculated to be ∼16.6 GHz^35^ around the laser wavelength of 1040 nm. This corresponds to a wavelength shift of ≈60 pm that lies within the spectral width of our FBG (200 pm). In order to show that SBS occurs in this laser cavity configuration, radio-frequency spectra have been acquired using a 43 GHz RF analyzer (Rohde&Schwarz FSW43) and are shown in Fig. 2 for laser operation well above threshold. We indeed observe two strong peaks around ∼16.2 and ∼16.6 GHz instead of a single one due to the propagation in two fibers (gain fiber and SMF) with different material properties and thus slightly different acoustic velocities. It is worth noting on Fig. 2(b) that these peaks are modulated at a frequency of ∼10 MHz, which corresponds to the free spectral range (FSR) of the cavity.

**Figure 3:**
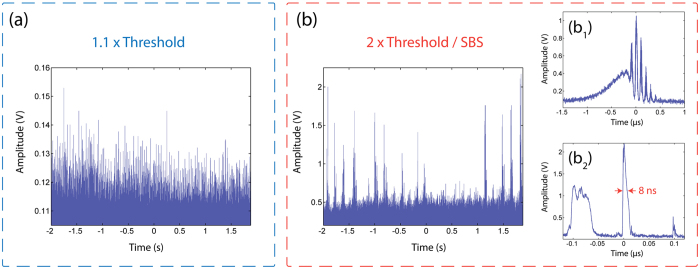
Time traces just above laser threshold (**a**) and at twice the threshold (**b**) showing the evolution to an unstable state. In (**a**) the typical events are in the *μ*s range. At twice the threshold, the corresponding pump intensity is above the Brillouin threshold. The insets in (**b**) show details of selected pulses with intensities exceeding the rogue wave intensity: (b_1_) at moderate intensity, the pulse exhibits typical partial mode-locking features, (b_2_) at higher intensities, the pulse breaks up into ns energetic pulses.

**Figure 4:**
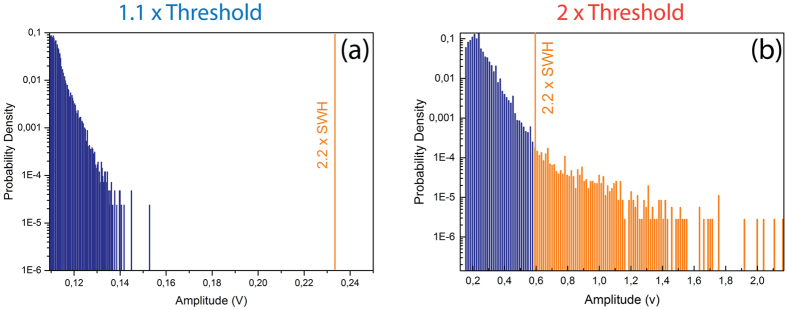
Probability density functions calculated from time traces presented in Fig. 3(a,b) and corresponding to respectively to 10^5^ and 8.10^5^ events detected. Events considered rogue, i.e. with intensities exceeding twice the SWH (solid vertical line) are denoted in orange.

Different indicators are generally used to attest of the emergence of rogue events in a physical system. First, they are defined through their specific statistical behavior with highly-skewed, “L-shaped” heavy-tailed intensity distributions. Another criterion, based on the oceanic analogy, is to calculate the significant wave height (SWH) which corresponds to the mean height of the highest third of waves^36^. An event is then considered extreme when its amplitude (or intensity in the case of optical events) exceeds twice the SWH. It is also possible to define this threshold through the calculation of the standard deviation *σ* as events exceeding the mean value plus 8*σ* will be considered as rogue^37^. Time series are shown on Fig. 3 for our laser system with two distinct dynamical behaviors observed for pump powers just above laser threshold (Fig. 3(a)) and at twice the threshold (Fig. 3(b)). The corresponding probability density functions (PDFs) are shown on Fig. 4.

Near threshold, the laser operates in a self-pulsing mode with pulse widths lying in the microsecond range (Fig. 3(a)). The intensity of the output pulses then exhibits a chaotic evolution with a PDF approximately following an exponential law as shown in Fig. 4(a). In this case, no event exceeding the “rogue wave intensity” (i.e. twice the SWH) is observed. On the contrary, when increasing the pump power to twice the laser threshold pump power and thus above the SBS threshold, we observe a completely different behavior as shown on Fig. 3(b). We indeed notice the emergence of bursts of short pulses on the trailing edges of the microsecond pulse envelopes, as depicted on Fig. 3(b1). These nanosecond bursts with 10 MHz repetition rate are typical of SBS buildup in a laser cavity. A selected giant pulse with intensity exceeding three times the rogue wave intensity is presented in Fig. 3(b2), where the pulse is shown to break up in several nanosecond-scale substructures. In this regime, the corresponding PDF is non-exponential with high-intensity events populating the tail of the distribution and considerably exceeding the rogue wave intensity, as represented on Fig. 4(b). Using the 8*σ* criterion (not represented here) yields approximately the same rogue wave intensity.

There is thus a dramatic change in the dynamics and statistics when increasing the pump power above the SBS threshold. The emergence of extreme events then seems to originate from the nonlinear spatio-temporal coupling between the laser and Stokes waves through SBS and cross-saturation coupling. The chaotic behavior of the laser operation, which acts as a pump for the Stokes wave, in conjunction with the stochastic nature of SBS seems to be at the origin of the emergence of rogue pulses with extreme amplitudes. In the cavity presented in Fig. 1, the FBG filtering allows for only one Stokes and one anti-Stokes orders to be resonant (i.e. under the gain bandwidth) and then to sufficiently interact with laser pulses. However, as cascaded anti-Stokes orders can easily be obtained in this kind of cavity^27^,^30^, we performed a second experiment by replacing the FBG with a broadband mirror, thus allowing not only two but several Stokes and anti-Stokes waves to be resonant in the cavity. Closely similar results are obtained indicating that the observed dynamics is not driven by the spectral filtering provided by the FBG. The main advantage of the FBG-based configuration is here to simplify the numerical model by considering only a single Brillouin order.

### Theoretical approach

**Figure 5:**
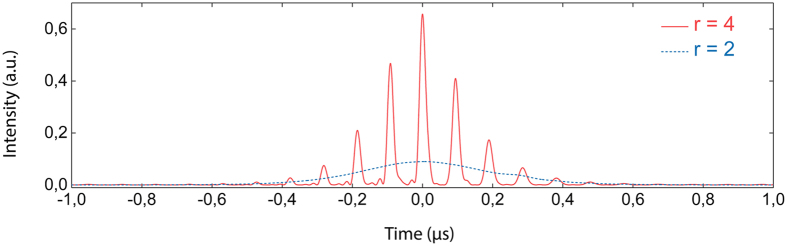
Detail of typical calculated pulses for different pump levels, near laser threshold (*blue dashed line*) and for a moderate pump power (*red solid line*).

The influence of stimulated Brillouin scattering in a high-loss linear laser cavity can be described with the coupled amplitudes model^38^. The latter is based on a rate-equation model including transitions such as stimulated and spontaneous emissions and as well as stimulated Brillouin scattering. The model thus involves six field equations for the forward and backward components of laser, Stokes and acoustic waves. This approach allows the description of phenomena occurring on a time scale well above the phonon lifetime. We consider here an inhomogeneously broadened gain medium included in a Fabry-Perot cavity therefore leading to two distinct population inversions related to the laser field and the Stokes wave, respectively. Both of them are coupled through the cross-saturation parameter^39^,^40^. A third matter equation is considered to account for the saturable absorption effect^41^,^42^ which can occur in the unpumped segment of the active fiber, especially at low pumping levels. It is worth noting that the introduction of a saturable absorption effect is crucial to numerically capture the laser dynamics observed near threshold. (See detailed model and parameters in Methods Section). Indeed, considering realistic laser parameters, our numerical model predicts irregular self-pulsing instabilities just above the laser threshold with pulse durations around 1 *μ*s, as shown in Fig. 5.

**Figure 6:**
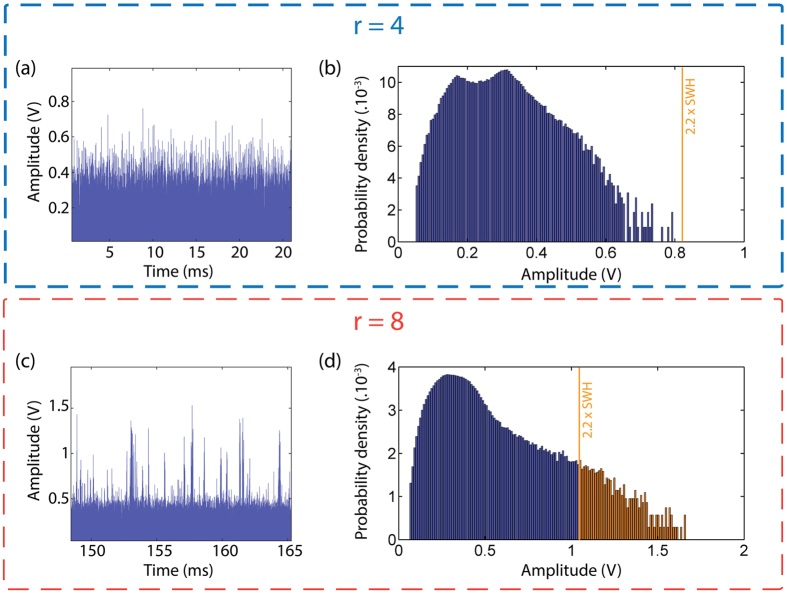
Time traces and histograms obtained using our numerical model for different pumping parameters. *r* represents the ratio of the pump power relative to the laser threshold power. (**a**,**c**) Selected times series showing the change in the dynamics and the emergence of bursts of pulses for a higher pumping rate. (**b**,**d**) Corresponding histograms. Changes in the probability density function are clearly visible as the distribution evolves from a nearly exponential to a skewed shape with a considerable amount of events exceeding the rogue wave intensity.

By increasing the pump level above the Brillouin threshold, which is estimated to be close to the laser threshold^38^, we observe the breaking of the microsecond pulses into regularly spaced short pulses with several nanoseconds duration and a period of 91.2 ns which is close to the inverse of the cavity FSR (see Fig. 5). In this case, the instability dynamics strongly changes and the emergence of high intensity short pulses randomly distributed in time is predicted, as evidenced by the laser output intensity shown in Fig. 6(a). However, at such moderate pump levels, the peak intensity statistics did not reveal any event exceeding the rogue wave intensity (Fig. 6(a,b)), in good agreement with the experimental observations. The peak intensity and the occurrence of these nanosecond bursts increase with pump power thereby allowing to reach and exceed the rogue wave intensity level at high pumping levels, as shown in Fig. 6(c,d). The corresponding probability density function then presents a considerable amount of events exceeding the rogue wave intensity.

## Discussion

Previous studies dealing with SBS-induced instabilities in fiber lasers have considered laser configurations where self-pulsing is initiated by stimulated Brillouin scattering. This is indeed the case in Erbium-doped fiber lasers where cw operation is generally observed at laser threshold and self-pulsing instabilities arise at high-pumping levels. Apart from the identification of large pulse-to-pulse intensity fluctuations, numerical and experimental analysis of laser dynamics did not reveal any rogue events in such laser platforms^29^. The situation is different in double-clad Ytterbium-doped fiber lasers featuring heavily-Ytterbium doped fibers. Indeed, our experiments reveal that self-pulsing arises at laser threshold and pulses with extreme intensities could be obtained at high pumping levels. They also suggest that the appearance of such extreme events is directly associated with the emergence of SBS-based instabilities. However, the existing theoretical models only based on the interaction between the laser and Stokes waves did not evidence the dynamics observed here^29^,^38^. The self-pulsing regime observed near threshold could then be attributed to the re-absorption of laser signal in the weakly-pumped section of the rare-earth-doped fiber^42^,^43^,^44^,^45^ or to ion-pairing acting as an effective saturable absorber^46^,^47^,^48^. By including an effective saturable absorption mechanism in the spatio-temporal model that fully takes into account the length dependence of the optical gain while also including the nonlinear effects within the active medium, we are now in a position to offer a plausible physical picture of the parameters driving the laser dynamics. Indeed, our numerical model predicts a self-pulsing operation mode with microsecond pulses near threshold while nanosecond transients with high-peak powers are obtained at higher pumping levels as in experiments. Statistical analyses finally reveal that the nonlinear interactions between the laser and Brillouin fields can lead to the emergence of high-intensity pulses with highly-skewed intensity distributions. It worth noting that the extreme events are numerically predicted for higher pumping levels than in experiments. This small discrepancy can be attributed to the simplified model used which did not account for the other nonlinear effects undergone by the laser pulses along the active and passive fibers such as stimulated Raman scattering and self-phase modulation. Moreover, the simplified model used to account for the saturable absorption mechanism can also explain this discrepancy to some extent. Indeed, our results show that the pumping level required to obtain extreme events in our laser system is decreased when increasing the strength of the saturable absorber (parameter b_1_, see Methods Section). The best matching between theory and experiments is for instance obtained for b_1_ = 5, a value which calls for more work to be physically justified.

In summary, we have presented experimental results demonstrating stochastic temporal dynamics and appearance of extreme events in a self-pulsing fiber laser. Using numerical simulations based on a coupled amplitudes model, we have then showed that the laser signal instability induced by an artificial saturable absorber is essential to explain the observed rogue dynamics. The resulting intensity fluctuations of the laser signal which constitute a pump for SBS are amplified thereby leading to the emergence of nanosecond bursts with extreme intensities. From this work, several questions are still opened such as the impact of other nonlinear effects, especially Raman scattering and self-phase modulation, as well as the possibility to fully control the dynamics through adapted cavity parameters. Even if this laser system is not an actual optical analogue of oceanic media, the complex nonlinear interactions involved represent a promising test-bed for studying the rich underlying physics involved in extreme events generation.

## Methods

The model involves three matter equations and six field equations, the forward and backward components of the laser and the Stokes and acoustic waves. The normalized model writes as follows:


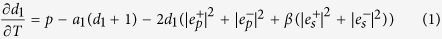



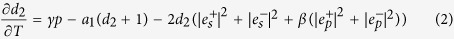



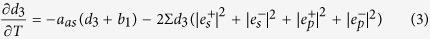



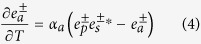



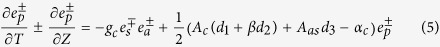



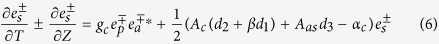


with the following boundary conditions (*R*_1_ > 99%, *R*_2_ = 4%): 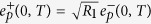, 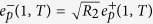, 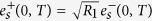 and 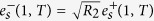, and: 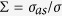, 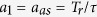, 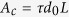, 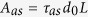, 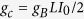, 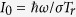, 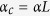, 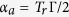, 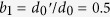.

*d*_1,2_ are the population inversions normalized with the concentration of active ions (*d*_0_ = 10^24^ m^−3^), *d*_3_ is the population inversion normalized with the concentration of saturable absorber 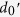. 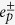, 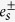 and 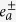 are the normalized laser, Stokes and acoustic field amplitudes, and the superscript plus and minus stand for the forward and backward components, respectively. The time *T* = *t*/*T*_*r*_ is normalized with the photon transit time along the fiber (*T*_*r*_ = 48 ns) and the longitudinal coordinate *Z* = *z*/*L* is normalized with the fiber length (*L* = 10 m) which is assumed to coincide with the total cavity length, so that the normalized cavity length is *l* = 1. The other parameters used in the simulation are given in Table 1. The cross-saturation parameter physically represents the strength of the coupling between the laser and Stokes fields through the amplifying medium. The laser threshold can be determined from the following expression:


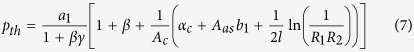


## Additional Information

**How to cite this article**: Hanzard, P.-H. *et al*. Brillouin scattering-induced rogue waves in self-pulsing fiber lasers. *Sci. Rep.* **7**, 45868; doi: 10.1038/srep45868 (2017).

**Publisher's note:** Springer Nature remains neutral with regard to jurisdictional claims in published maps and institutional affiliations.

## Figures and Tables

**Table 1:** Parameters used in the numerical simulations.

*g*_*B*_ = 1.66 × 10^−11^ *m*/*W*	Brillouin gain
*g*_*c*_ = 100	Normalized Brillouin gain
Γ = 625 × 10^5^ s^−1^	Acoustic wave damping coefficient
*τ* = *τ*_*as*_ = 800 *μs*	Population inversion lifetime
*σ* = 32 × 10^−25^ m^2^	Emission cross-section
*σ*_*as*_ = 32 × 10^−25^ m^2^	Saturable absorber cross-section
*α* = 0.0458 m^−1^	Absorption coefficient
*p*	Pumping parameter (assumed uniform along the fiber)
*r* = *p*/*p*_*th*_	Ratio of the pump power relative to the laser threshold power
*γ* = 0.7	Dichroism in the pumping process (laser anisotropy)
*β* = 0.5	Cross-saturation parameter
*R*_1_ and *R*_2_	Reflection coefficients (FBG and cleaved fiber)
